# Research should remain a priority in 21st century radiology recruitment to training

**DOI:** 10.1259/bjr.20221083

**Published:** 2023-02-20

**Authors:** Paul Jenkins, Indrajeet Mandal, Jim Zhong, Vicky Goh

**Affiliations:** 1 Department of Radiology, University Hospital Plymouth NHS Trust, England, United Kingdom; 2 Peninsula Radiology Academy, Plymouth, UK; 3 Department of Radiology, Oxford University Hospitals, Oxford, United Kingdom; 4 Leeds Institute of Medical Research, University of Leeds, Leeds, UK; 5 Department of Radiology, Leeds Teaching Hospitals NHS Trust, Leeds, United Kingdom; 6 School of Biomedical Engineering and Imaging Sciences, King’s College, London, United Kingdom; 7 Department of Radiology, Guy’s & St Thomas' NHS Foundation Trust, London, United Kingdom

## Abstract

Research drives innovation, however, recently Clinical Radiology has been overwhelmed by increased clinical demand, workforce shortages and lack of funding/protected research time. The newly released 2023 radiology speciality application process gives research a lower priority compared to other domains such as audit which is concerning given the current lack of research culture within the speciality. It is vital for the future radiology workforce to engage with research and in order to fulfil the Royal College of Radiologist’s new curriculum aims of strengthening research within training, we must continue attracting the brightest and best candidates and ensure research remains a priority.

## Background

Radiology is a fast moving speciality driven by advances in technology.^1^ However, the speciality continues to lag behind others with respect to clinical involvement in research, due to a number of factors. These include a lack of capacity for research activity in overwhelmed clinical departments; a lack of time for radiologists to support clinical research with ongoing workforce shortages; and lack of structure and funding to undertake research compared to other specialities, all contributing to a lack of research culture.^2,3^ The number of radiology academics is low compared to other clinical specialities, with few radiologists listed within the National Institute for Health and Care Research senior investigator directory.^4,5^ Consequently, much of the imaging research in the UK are undertaken by non-radiologists.

Recent publications have emphasized the barriers to research for diagnostic^6^ and interventional radiology trainees.^7^ Whilst the latest 2021 RCR curriculum^8^ highlights the importance and requirement for research within training, research culture change within radiology has some way to go. Indeed, we write to express our concern regarding the priority that research has been given within the new radiology application selection criteria for the 2023 Clinical Radiology and integrated IR - Clinical Radiology (Intervention) - ST1 training positions.^1^

## New radiology application process

The new 2023 application process portfolio^8^ scoring reflects seven domains: commitment to speciality, leadership, teaching, teaching qualification, audit and quality improvement (QI), research and prizes. The domains are scored variably with a total of 45 points available, and a maximum of five points awarded for research. This is compared to a maximum of seven points for audit and QI, a maximum of ten points for teaching across two sections and a further seven points for leadership. Achieving a PhD, the pinnacle of research qualifications (five points), is valued less than a prize from a national society (six points) and equal to being involved at committee level with a regional radiology society, such as an undergraduate radiological society. Furthermore, a first author radiology-related paper is considered equal to a contribution to a regional teaching programme and of less value than a single-closed-loop audit.

## Discussion

We recognise the importance of education, audit and quality improvement in medicine. However, research forms the bedrock of evidence-based practice and it should not be undervalued. We welcome the high scoring across domains, rewarding national leadership, quality teaching and clinical excellence along with the commendable desire to reward commitment to speciality. But, excellence in research should be similarly rewarded, given the time, effort and personal commitment to achieve good quality research. Our concern is that this scoring system will likely impact on how future radiology applicants prepare their curriculum vitae. Potential negative effects include fewer prospective radiologists engaging in radiology research at an early-career stage and lack of research skills across the incoming workforce. This comes at a time when the UK radiology and specifically IR academic infrastructure lags behind other specialties and other nations. A study from the Royal College of Surgeons, UK, which surveyed 848 trainee surgeons, found up to 68% of trainees had completed a postgraduate degree.^9^ This was stark contrast to the 11.3% of the UK IR trainee cohort who had a postgraduate qualification and up to 25% of IR trainees in European countries.^10^ Kamaldeen et al sampled research interested UK radiology registrars and demonstrated that only 25.9% had a higher degree prior to enter radiology training with only 4.5% obtaining a higher degree during training. Even within a research focused group, the proportion is markedly lower compared to surgical counterparts.^6^

We have lessons to learn from the USA in terms of the stronger research infrastructure available in many of their IR departments. Since the introduction of a highly competitive integrated IR residency programme, there have been higher calibre applicants to IR with the mean number of academic achievements (including abstracts, presentations and publications) for the average successful IR applicant being 12.2 compared to 8.1, the average for US applicants.^11^ Within the USA, over 10% of IR applicants had two or more publications yet were unsuccessful with obtaining a training place.

Medical students have needed ways to standout amongst their peers, one of which is through academic achievements.^12^ This has led to the formation of resident-led (*i.e.,* registrar-led) medical student interventional radiology research interest groups to help give students more research opportunities. A UK IR trainee-led research^13^ collaborative was also set up in 2021 to encourage more medical students and junior doctors to be involved in research however without crediting such achievements at these key entry points into the speciality, we fear such endeavours will fail.^14^

Evidence also suggests that career development and a diversified professional role, which enables the acquisition of new knowledge and skills, was a determinant for happiness.^15^ The opportunity to acquire higher qualifications whilst building a clinical-academic career could contribute to greater job satisfaction for future radiologists. It is vital that academically minded junior doctors are encouraged to pursue careers in radiology for the potential to also develop such careers.

Whilst the 2021 RCR curriculum^8^ does strengthen clinical radiology’s commitment to research within training, we need to continue to attract high calibre candidates with the right skillset for radiology. Seemingly deprioritising research at the point of entry into the speciality is not the right approach.

## References

[1] NHS Health Education . Clinical Radiology ST1 Self-Assessment. 2023. Available from: https://www.oriel.nhs.uk/Web/Vacancy/VacancyDetails

[2] Makris GC, Burrows V, Lyall F, Moore A, Hamady MS . Vascular and interventional radiology training; international perspectives and challenges. Cardiovasc Intervent Radiol 2021; 44: 462–72. doi: 10.1007/s00270-020-02688-y 33174143

[3] Rodrigues J, O’Regan T, Darekar A, Taylor S, Goh V . National institute for health and care research (NIHR) imaging workforce group, NIHR imaging steering group, and NIHR clinical research network imaging champions. current pressure on the UK imaging workforce deters imaging research in the NHS and requires urgent attention. Clin Radiol 2022; 00515–3. 10.1016/j.crad.2022.07.015 36167569

[4] National Institute for Health Research. NIHR Senior Investigators 2021. Available from: at https://www.nihr.ac.uk/documents/nihr-senior-investigators-2021/26864 Accessed 14/11/22

[5] National Institute for Health Research. Senior investigator Directory 2022. Available from: https://si.gmg-is.co.uk/home

[6] Kamaledeen S, Brown P, Gangi A, Pantelidou M, Chan N . Survey of research participation amongst UK radiology trainees: aspirations, barriers, solutions and the radiology academic network for trainees (radiant). Clin Radiol 2021; 76: 302–9: S0009-9260(21)00060-X. doi: 10.1016/j.crad.2021.01.005 33583566

[7] Jenkins P, MacCormick A, Harborne K, Liu W, Mahay U, Zhong J, et al . Barriers to research in interventional radiology within the UK. Clin Radiol 2022; 77: e821–25: S0009-9260(22)00641-9. doi: 10.1016/j.crad.2022.08.146 36216606

[8] RCR . Clinical Radiology Curriculum. 2021. Available from: https://www.rcr.ac.uk/clinical-radiology/specialty-training/curriculum/clinical-radiology-curriculum

[9] O’Callaghan J, Mohan HM, Sharrock A, Gokani V, Fitzgerald JE, Williams AP, et al . Cross-Sectional study of the financial cost of training to the surgical trainee in the UK and ireland. BMJ Open 2017; 7: e018086: e018086. doi: 10.1136/bmjopen-2017-018086 PMC5695344 29146646

[10] Makris GC, Burrows V, Lyall F, Moore A, Hamady MS . Vascular and interventional radiology training; international perspectives and challenges. Cardiovasc Intervent Radiol 2021; 44: 462–72. doi: 10.1007/s00270-020-02688-y 33174143

[11] National resident matching program. charting the outcomes 2022 report. 2021. Available from: at https://www.nrmp.org/wp-content/uploads/2022/07/Charting-Outcomes-MD-Seniors-2022_Final.pdf accessed 23/12/22

[12] Khaja MS, Jo A, Sherk WM, Majdalany BS, Dunnick NR, Bailey JE, et al . Perspective on the new IR residency selection process: 4-year experience at a large, collaborative training program. Acad Radiol 2022; 29: 469–72: S1076-6332(21)00056-8. doi: 10.1016/j.acra.2021.01.028 33602595 PMC8803050

[13] Som A, Lang M, Di Capua J, Chonde D, Cochran R. Resident-Led Medical Student Radiology Research Interest Group: An Engine for Recruitment, Research, and Mentoring—Radiology In TrainingRadiology 2021 300:1, E290-E292 10.1148/radiol.2021204518 33944632

[14] Mandal I, Zhong J, Borchert R, Keni S, Jenkins P, MacCormick A, et al . The unite collaborative: early experiences of introducing collaborative trainee research to interventional radiology in the United Kingdom. Cardiovasc Intervent Radiol 2022; 45: 259–60. doi: 10.1007/s00270-021-02984-1 34668056 PMC8525613

[15] Muthuri RNDK, Senkubuge F, Hongoro C . Determinants of Happiness among healthcare professionals between 2009 and 2019: a systematic review. Humanit Soc Sci Commun 2022; 7: 98. doi: 10.1057/s41599-020-00592-x

